# Impact of process interference on memory encoding and retrieval processes in dual-task situations

**DOI:** 10.3758/s13421-024-01539-2

**Published:** 2024-03-04

**Authors:** Sandra Hensen, Iring Koch, Patricia Hirsch

**Affiliations:** Cognitive and Experimental Psychology, Institute of Psychology, RWTH Aachen University, Jaegerstrasse 17/19, 52066 Aachen, Germany

**Keywords:** Memory, Encoding, Retrieval, Dual-task, Processing conflict

## Abstract

Dual-tasks at the memory encoding stage have been shown to decrease recall performance and impair concurrent task performance. In contrast, studies on the effect of dual-tasks at the memory retrieval stage observed mixed results. Which cognitive mechanisms are underlying this dual-task interference is still an unresolved question. In the present study, we investigated the influence of a concurrent reaction-time task on the performance in a long-term memory task in two experiments. In Experiment 1, participants performed an auditory-verbal free recall memory task and a visual-manual spatial Stroop task in a single or dual-task condition, either at the encoding or retrieval stage of the memory task. In Experiment 2, we examined the influence of processing conflicts in a concurrent RT task on memory encoding. Both experiments showed detrimental effects on recall accuracy and concurrent RT task performance in dual-task conditions at the encoding stage. Dual-task conditions at the retrieval stage led to a slowdown in recall latency and impaired concurrent RT task performance, but recall accuracy was maintained. In addition, we observed larger Stroop congruency effects in the dual-task conditions, indicating an increased processing conflict. However, in Experiment 2, we analyzed the effect of the processing conflict in a time-locked manner and could not find a significant influence on success of memory encoding. These findings suggest that processes in both tasks share the same limited capacity and are slowed down due to parallel processing, but we could not find evidence that this is further influenced by task-specific processing conflicts.

In our modern technology-driven working environments, multitasking has become increasingly common. For example, health-care studies have identified multitasking as a frequently used strategy for physicians to handle competing demands under time pressure (Heng, 2014). Especially during patient handoffs in polytrauma care, physicians not only have to take care of patients, attend to the electronic records, and anticipate as well as decide which actions they need to do next, but listen simultaneously to the verbally transmitted patient-related information or transmit information themselves. However, this can lead to negative consequences such as communication errors and information loss, which might be harmful for patients. Evans et al. (2010) showed that only 75% of information handed over by paramedics to the trauma team was documented, and only 67% of information from in-hospital handovers. Thus, about 30% of information was lost and potentially not available for subsequent instances. The aim of the present study was to examine the underlying cognitive mechanisms of this information loss. To this end, we investigated the influence of multitasking on encoding and retrieval of auditory verbal information from long-term memory in two experiments.

Researchers in cognitive psychology have long studied the relationship between multitasking and memory. In multitasking situations, cognitive processes of multiple tasks overlap in time (Koch et al., 2018). A typical multitasking situation is, for example, a dual-task paradigm in which two or more tasks are performed simultaneously (i.e., dual-task condition). Task performance is also measured in isolation (i.e., single-task condition), and compared with dual-task performance (see Fischer & Janczyk, 2022; Pashler, 1994, for reviews). A performance decline in dual-task conditions relative to single-task conditions is referred to as dual-task costs. Dual-task interference has been investigated at the stage of memory encoding as well as memory retrieval.

Dual-tasks at the memory encoding stage showed large decrements in later recall and recognition memory performance in dual-task compared with single-task conditions with a variety of different concurrent tasks (e.g., card sorting, digit monitoring or reaction time [RT] tasks; see, e.g., Anderson & Craik, 1974; Baddeley et al., 1984; Craik et al., 1996; Fernandes & Moscovitch, 2000; Naveh-Benjamin et al., 2000a). In addition, simultaneously encoding words resulted in poor concurrent task performance (e.g., Craik et al., 1996; Fernandes & Moscovitch, 2000).

The size of these dual-task costs is influenced by different variables, such as task complexity (e.g., Naveh-Benjamin et al., 2000a). For example, Naveh-Benjamin et al. (2000a) examined the effects of decision and motor difficulty of a visual-manual concurrent RT task on memory encoding in an auditory-verbal cued recall memory task with unrelated word pairs. In the RT task, rectangles were visually presented in horizontally arranged boxes on a screen, and participants were asked to manually press corresponding keys, which caused the rectangle immediately to move to another random box. Complexity was increased by varying the number of response alternatives (three-choice vs. six-choice) or asking participants to press each key either once or twice. Results showed that the more complex the decision component was during encoding (i.e., increasing number of response alternatives), the more it impaired later cued recall performance. An increase in motor difficulty of the concurrent task (i.e., pressing each key more than once) had no negative effect on memory performance. Thus, when interpreting dual-task interference, it is important to consider which combination of primary and concurrent task was used.

Dual-task costs at memory encoding have been explained by a capacity limitation of attentional resources. In detail, researchers proposed that attentional resources are utilized simultaneously for both tasks, and a decline in performance will occur when the tasks require more capacity than is available (e.g., Craik et al., 1996; see also Kahnemann, 1973). Further, attention control and working memory (WM) capacity have been identified as crucial constructs for variance in dual-task performance (Redick et al., 2016). Attention control is defined as the general ability to engage in and manage goal-directed behavior by maintaining goal-relevant information and filtering or inhibiting irrelevant information (i.e., attention control regulates maintenance and disengagement processes; e.g., Draheim et al., 2022). The working memory is considered a capacity-limited system that is responsible for temporarily maintaining information for use in ongoing tasks with separate domain-specific modules (visuo-spatial sketchpad for visual information and phonological loop for auditory information) and a central executive as supervisory system primarily responsible for coordinating activity (Baddeley & Hitch, 1974). However, which specific cognitive mechanisms are underlying these dual-task costs at memory encoding is still an unresolved question (Naveh-Benjamin et al., 2014).

Research using the psychological refractory period (PRP) paradigm could give some insight into understanding the underlying cognitive mechanisms. In the PRP paradigm, two RT tasks are performed in a dual-task condition, but with varying temporal overlap between stimulus presentations (i.e., different stimulus-onset asynchrony; SOA). The PRP effect describes the phenomenon that when the two stimuli are presented shortly after each other (i.e., short SOA), the response to the second stimulus is slowed down, and thus more dual-task interference can be observed relative to situations with a long SOA (e.g., Pashler, 1994; Welford, 1952, for reviews). One possible explanation for the PRP effect is that there is a processing bottleneck during response selection (see Koch et al., 2018, for a review). According to this explanation, processes that require “central” capacity (i.e., decision and response selection processes; e.g., Fagot & Pashler, 1992; McCann & Johnston, 1992) are capacity-limited and thus cause a bottleneck when two tasks draw upon the same processing resource simultaneously (Pashler, 1994).

Later studies, using a PRP-like paradigm, showed a similar type of dual-task interference when memory encoding tasks were combined with RT tasks (e.g., Jolicoeur, 1999a, 1999b; Jolicoeur & Dell’Acqua, 1998; Koch & Jolicoeur, 2007; Koch et al., 2003; Koch & Prinz, 2002; Koch & Prinz, 2005). For example, Jolicoeur and Dell’Acqua (1998) presented one or three characters (letters or symbols) visually on a screen, followed by an auditory stimulus for a speeded two-choice RT task. When participants were asked to report the visual stimulus after response execution in the auditory task, RTs to the auditory stimulus slowed down relative to when the visual stimulus could be ignored. This dual-task interference further increased when participants needed to encode more characters and when the SOA between both the visual and auditory stimuli was reduced. The authors suggested that encoding information into short-term memory also requires “central” processing, like response selection processes. Thus, because of a capacity limitation, processes either must wait or they slow down (see also Koch & Prinz, 2002). In line with this conclusion, Jolicoeur (1999a) showed in another study that accuracy for a visual memory task was decreased when the visual stimulus was encoded shortly after the presentation of an auditory stimulus, which required a speeded choice reaction (see also Jolicoeur, 1999b).

In contrast to dual-tasks at the memory encoding stage, early studies investigating the effects of dual-task demands during memory retrieval showed no or only small dual-task costs in the memory task (e.g., Baddeley et al., 1984) and only concurrent task performance was significantly impaired (e.g., Craik et al., 1996; Naveh-Benjamin et al., 2000b). Hence, it was first assumed that memory retrieval processes are in some sense resistant to dual-task interference, but that such resilience requires considerable effort (i.e., resulting in poor concurrent task performance). Later studies could not confirm this conclusion and found clear negative effects on both memory and concurrent task performance (e.g., Lozito & Mulligan, 2006; Rohrer & Pashler, 2003), indicating that the exact combination of primary and secondary tasks is also important for dual-tasks at memory retrieval. Yet, in terms of the recall performance, most studies found larger dual-task costs at the stage of memory encoding compared with memory retrieval (see, e.g., Naveh-Benjamin et al., 2006).

Rohrer and Pashler (2003) argued that a potential reason for the lack of finding dual-task interference on memory retrieval processes could be that the concurrent tasks used in these early studies (e.g., card sorting in Baddeley et al., 1984) placed insufficient demands on “central” processes. To test the role of central processes in dual-task interference, Rohrer and Pashler (2003) developed a RT task in which participants should devote a substantial proportion of their time to response selection processes while simultaneously recalling previously to-be-remembered words. In their visual-manual serial-choice RT task, different colored rectangles were presented with a constant interstimulus interval whose duration was determined by subject’s prior performance (i.e., the temporal density was high). In addition, the authors included an analysis of the time course of memory retrieval, the so-called recall latency, and suggested that a null effect on recall total (i.e., the number of correctly recalled items) does not ensure a null effect on recall latency. For recall latency, the latency of each recalled word equals the time elapsed between the beginning of the recall period and the vocal onset of the response. Mean recall latency of a participant simply equals the arithmetic mean of these latencies. For example, if a participant recalled three words with latencies of 5, 10 and 30 seconds, mean recall latency would be 15 sec ((5 + 10 + 30)/3). Results showed that recall total as well as recall latency were impaired in the dual-task condition, suggesting that task complexity plays an important role in dual-task interference at memory retrieval.

Besides task complexity, the degree to which the primary memory task and concurrent task rely on overlapping processing resources affects dual-task costs at memory retrieval (e.g., Fernandes & Moscovitch, 2000, 2002, 2003; Skinner & Fernandes, 2008). For example, Fernandes and Moscovitch (2000) demonstrated in a series of experiments that different concurrent tasks with either verbal or nonverbal stimulus material (e.g., word and digit monitoring) had different effects on memory encoding and retrieval processes. In a cued recall memory task with unrelated word pairs, all concurrent tasks (with verbal and nonverbal stimulus material) led to large dual-task costs during memory encoding in the memory and concurrent task. Dual-tasks at memory retrieval only led to interference when the concurrent task involves verbal stimulus material as in the memory task. Accordingly, the authors concluded that encoding competes for general resources, thus any task that draws resources away also decreases memory performance. In contrast, dual-task costs in memory retrieval are influenced by the degree to which both tasks rely on overlapping processing resource (i.e., worse performance when both tasks included verbal processing compared with numerical processing in the concurrent task).

In sum, dual-task costs have been observed at the memory encoding as well as retrieval stage. Task complexity and the degree to which the memory and concurrent task rely on overlapping processing resources have been identified as crucial mediating factors for the size of those dual-task costs, but which cognitive mechanisms are underlying these negative effects is still not answered. Previous studies using the PRP paradigm suggested that the source of dual-task interference could be allocated at the “central” processing stage. In the present study, we aim to further investigate the role of general load at the “central” processing stage and task-specific interference on memory processes.

## The present study

In the current research, we used a dual-task paradigm with a memory task and speeded RT task to (1) reexamine the previously described negative effects of dual-task demands at memory encoding and retrieval on task performances, (2) examine how overlapping processing resources between both tasks alters these dual-task demands, and (3) investigate whether processing conflicts in a concurrent RT task modulate the success of memory encoding. The goal is to gain insight into the cognitive mechanisms underlying the dual-task interference between memory and response selection processes.

In our experiments, we used auditory-verbal free recall memory tasks with clearly separated encoding and retrieval phases as primary task. Participants needed to encode auditory word lists and verbally recall the to-be-remembered words after performing a distractor activity. As concurrent task, we employed a visual-manual four-choice spatial Stroop task that was performed in the encoding or retrieval phase of the primary memory task. In this task, participants need to classify the spatial meaning of either symbolic arrow stimuli or verbal word stimuli, which are presented in an irrelevant position on the screen. The spatial meaning and spatial position of the stimulus either matched (congruent trial) or mismatched (incongruent trial). Performance is typically worse in incongruent trials compared with congruent trials (Stroop congruency effect; see Lu & Proctor, 1995, for a review). More specifically, in congruent trials, the meaning and position of the stimulus activate the same response (e.g., the word “left” on the left side of a screen activates a left key press). In contrast, in incongruent trials, they activate competing responses (e.g., the word “left” on the right side of a screen activates both a left and right key press), resulting in a processing conflict at target identification and response selection (Botvinick et al., 2001).

For the primary memory task, we only used two- and three-syllable words to control for the word length effect (Baddeley et al., 1975). The word length effect describes that it is easier to recall sequences of short words compared with long words. This is because it takes longer to rehearse and produce long words during recall and thus, this allows more time for the memory trace to deteriorate. In addition, to increase the transferability of our results into applied contexts where people mostly need to process and remember specific information from their working field (e.g., medical scenarios), we decided to use auditory stimulus material that is psychology-related (like names, symptoms from mental illnesses, and psychological constructs).

A spatial Stroop task was chosen as concurrent RT task, because we wanted to have a cognitive demanding task where participants needed to devote a substantial proportion of their time to response selection processes (as suggested by Rohrer & Pashler, 2003). Again, the idea was to increase transferability of the results to applied contexts, like polytrauma care, where people need to perform cognitively demanding visual-manual tasks while listening to important patient-related information. Note that even if in those applied contexts RT differences in milliseconds do not play a crucial role, we are still able to draw a conclusion about the underlying cognitive mechanisms of the dual-task interference from the RT task, which in turn can help us to understand the reason for information loss in those applied scenarios. Lastly, in the first experiment, we decided to use either symbolic arrows or verbal words as stimuli in the RT task to determine the effect of overlapping processing resources (in terms of processing codes; symbolic vs. verbal and verbal vs. verbal) between both tasks on performances.

## Experiment 1

Experiment 1 examined the impact of dual-task demands at the memory encoding and retrieval stages on memory and concurrent RT task performances. Further, the impact of overlapping processing resources in both tasks was investigated. We presented participants with lists of words, which they needed to recall in a later retrieval phase in single- or two different dual-task conditions (dual-task at memory encoding and memory retrieval). In the dual-task conditions at memory encoding or retrieval, a visual-manual concurrent RT task with either symbolic arrows or verbal words as stimuli was performed simultaneously (included as between-subjects factor; i.e., there were two different groups). Performance of the RT task was also measured in isolation. We used a digit span task to control for differences in the number of items that participants can maintain and recall in the two different groups (arrow vs. word group; see Baddeley, 2000; Ramsay & Reynolds, 1995, for a review of the digit span task^1^).

For the dual-task condition at the memory encoding stage, we hypothesized both a large decline in memory accuracy and a decreased performance in the concurrent RT task compared with the single-task condition. For the dual-task condition at the memory retrieval stage, we predicted a smaller decrement in memory accuracy compared with encoding, but still a worse performance in the concurrent RT task compared with the single-task condition. Additionally, for memory retrieval, we hypothesized that the decrement in memory accuracy is larger when verbal words are used as stimulus material in the concurrent RT task compared with symbolic arrows. Further, we expect a Stroop congruency effect in the concurrent RT task.

### Method

#### Stimuli, tasks, and responses

As primary task, participants performed an auditory-vocal free recall memory task, including a distractor activity (counting backwards by threes). As concurrent RT task, participants performed a visual-manual spatial Stroop task, in which they classified either the direction of an arrow (arrow group) or the meaning of a location word (word group) while ignoring the position of the target stimulus (arrow or word) on the screen.

The auditory stimulus material for the free recall memory task consisted of 150 German two- or three-syllable words, which were generated with a text-to-speech converter. The words were mainly selected from the *Diagnostic and Statistical Manual of Mental Disorders* (American Psychiatric Association, 2013; e.g., “Depression,” “Emotion,” “Memory”), so that they were psychology related. A female voice was selected, and the duration of each word was adjusted to 1 second. The words were randomly assigned to lists, without mixing the two- and three-syllable words and no word appearing in more than one list. In total, there were nine lists with 15 words and three lists with five words. The word order for all lists was randomized for each participant. A 600-Hz “beep” sound was used as a signal tone that cues the start of the retrieval phase of the memory task. All auditory stimuli were presented binaurally via headphones (Sennheiser HD-200 Pro).

The visual stimulus material for the RT task (i.e., spatial Stroop task) consisted of a fixation cross (+), the German location words “LINKS,” “RECHTS,” “OBEN,” and “UNTEN” (*left*, *right*, *up*, and *down*) or arrows pointing left, right, up and down. All visual stimuli were presented in white on a black 24-inch screen, placed at a distance of approx. 70 cm. Except for the arrows, all visual stimuli were displayed in Arial font with a height of 4 cm; the size of the arrows was 4 × 8 cm. As an error feedback, the German word “FEHLER” (error) was displayed. The fixation cross and error feedback were presented in the screen center. The position of the words “LINKS” and “RECHTS” or the left- and right-pointing arrows changed horizontally (50:50), with 4 cm to the left or right side of the screen center (e.g., the word “LINKS” or the left-pointing arrow was displayed half the time on the left side and half the time on the right side of the screen). The position of the words “OBEN” and “UNTEN” or the up- and down-pointing arrows changed vertical (50:50), positioned 4 cm above or below the screen center (e.g., the word “OBEN” or the up-pointing arrow was displayed half the time in the upper part and half the time in the lower part of the screen). Thus, 50% of the trials were congruent and 50% of the trials were incongruent.

Vocal responses (free recall of the auditory stimuli and counting backwards for the distractor task) were recorded via microphone. Manual responses (for the RT task) were made via a QWERTZ keyboard by pressing “Y” for the response left, “X” for the response right, arrow key “up” for the response up and “down” for the response down with the middle and index finger of the left and right hand. The experiment was programmed and run using PsychoPy 3 (Version 2021.1.4; Peirce et al., 2019) on a Windows 10 computer.

#### Procedure

The experiment took approx. 45 minutes. Instructions were given in both verbal and written form, emphasizing speed and accuracy in all tasks. Participants were randomly assigned to one of two different groups (arrow group vs. word group), but the number of participants in each group was held constant. Before the main experiment started, participants filled out a demographic questionnaire and performed a digit span task. A *t* test showed no difference in total digit span between both groups (*M* = 7.17 [arrow group] vs. *M* = 6.96 [word group];* t* < 1). The main experiment consisted of two different single-task conditions (memory task and RT task) and two different dual-task conditions (at the encoding stage and retrieval stage of the memory task).

The memory task included an encoding phase, a distractor activity, and a retrieval phase (free recall). In each block during a 60-sec encoding phase, 15 words were presented serially via headphones at a rate of one per 4 sec, and a fixation cross was displayed on the screen the whole time. In a following 30-sec distractor activity, participants verbally counted backwards by threes from a randomly chosen three-digit number that appeared on the screen. After that, a signal tone (500 ms) cued the beginning of a 60-sec recall period, in which participants were instructed to recall the most recent list of 15 words verbally in any order.

In the RT task, each trial started with the presentation of a fixation cross and location stimulus (either an arrow or location word) for up to 1,500 ms. The location stimulus disappeared after response execution or time-out (no response within 1,500 ms) and the fixation cross remained for a variable response–stimulus interval (RSI) of 2,000 ms minus RT, to keep the time of one trial constant (2000 ms). In case of an error or time-out, a visual error feedback was displayed for 300 ms, followed by the fixation cross for 1,700 ms minus RT (or minus 1,500 ms in case of a time-out). The stimulus presentation was random with the restriction that all stimuli were displayed equally often. Participants performed 30 trials (15 congruent and 15 incongruent trials) in each block.

For the dual-task parts, the RT task was performed during either the encoding phase or the recall phase of the memory task. In the dual-task at memory encoding condition, participants needed to respond to two Stroop stimuli during the encoding of one word (15 words in the memory task and 30 Stroop trials in total per block). The distractor activity and retrieval phase remained the same as in the single-task condition of the memory task. In the dual-task at memory retrieval condition, participants were asked to perform the RT task while simultaneously recalling the most recent word list (60-sec recall period and 30 Stroop trials per block). Again, the encoding phase and the distractor activity remained the same as in the single-task condition of the memory task.

To become familiar with the experimental tasks, participants first performed a practice block of the RT task with 16 trials, followed by a practice block of the memory task with a five-element word list in the encoding phase (20 sec), a 15-sec distractor activity, and a 20-sec recall period. Further, practice blocks for the two dual-task conditions were conducted with five-element word lists in the memory task and 10 trials of the RT task in either the encoding phase or recall period. There were three experimental blocks for each of the four conditions (single-task memory task and single-task RT task; dual-task at the memory encoding stage and dual-task at the memory retrieval stage; 12 experimental blocks in total), which were counterbalanced with a Latin square design across participants in each group. The order of the nine different word lists used in the single- and dual-task blocks of the memory task was also counterbalanced across participants in each group.

#### Design

For analysis of the primary memory task, we used a 3 × 2 mixed factorial design. The independent within-subject variable was task load (single-task vs. dual-task at encoding vs. dual-task at retrieval) and the independent between-subject variable was stimulus material (arrow vs. word group). The dependent variables were recall total and recall latency.

For the concurrent RT task, the independent within-subject variables were task load (single-task vs. dual-task at encoding vs. dual-task at retrieval) and congruency (congruent vs. incongruent) and the independent between-subject variable was stimulus material (arrow vs. word group). The dependent variables were RT and error rates.

#### Participants

Forty-eight psychology students (32 female; mean age = 23.22 years, *SD* = 3.99) took part in the experiment. They were prescreened according to their age (between 18 and 35 years) and native language (German). All gave their informed consent for participation, reported normal or corrected-to-normal vision and hearing acuity, and received partial course credit or monetary compensation (10€ per hour) in exchange. Nine additional participants were tested, but their data could not be analyzed due to exclusion criteria (see Results).

To determine the sample size, a power analysis was conducted using MorePower 6.0 (Campbell & Thompson, 2012). Power was calculated for an analysis of variance (ANOVA) with two within-subject factors (one with two and one with three levels) and one between-subjects factor (two levels). As effects of interest, all three main effects (task load, congruency, and stimulus material) were selected, based on our most important hypotheses. With a medium to large effect size (η_p_^2^ = .10) and an alpha of 0.05, the power analysis showed that at least 48 participants were required to achieve a power of 0.8.

### Results

The data were analyzed with frequentist and Bayesian analysis, using IBM SPSS and the BayesFactor package for R (Rouder et al., 2017). Therefore, *p* values, effect sizes, and Bayes factors (BF) are reported. For the frequentist ANOVA and *t* tests, all analyses were calculated at α = .05. For the Bayesian analysis, BF_10_ gives evidence in favor of the alternative hypothesis over the null hypothesis and BF_01_ in favor of the null hypothesis over the alternative hypothesis. BF_10_ or BF_01_ values between 3 and 20 indicate positive evidence, between 20 and 150 strong evidence, and greater than 150 very strong evidence in favor of the alternative or null hypothesis (Kass & Raftery, 1995).

BFs were calculated in two steps. First, for each set of independent variables, models were built with all the possible combinations of effects involving them (e.g., for a design with the independent variables A and B, the following models were built: null model without any effects, model with only main effect of A, model with only main effect of B, model with main effects of A and B, but no interaction, full model with main effects of A and B and interaction A × B). After the models were built, and the likelihood for each model given the data were computed, BFs were calculated as the ratio between this likelihood and that of a null model containing only the grand average and participants as a random effect. Afterwards, inference on a particular effect of interest was achieved by comparing the BF of the best fitting model (i.e., that with the highest BF compared with the null model) with that of an identical model which differs only in the presence/absence of the effect of interest (Rouder et al., 2017).

All practice blocks were discarded for all analyses. For the recall latency analysis of the memory task, the voice onset of each word was extracted from the recorded files, and latencies of both incorrect responses and false alarms (such as a cough or an errant word) were removed from the data. For the RT analysis of the concurrent RT task, trials with an erroneous response (6.30%) and trials deviating more than ±3 standard deviations from each participant’s individual mean RT per condition (1.10%) were additionally eliminated from the remaining data.

Participants’ data sets were excluded from analyses when they recalled zero words in one or more block(s) of the memory task or their error rate was above 40% in one or more block(s) of the concurrent RT task (*n* = 9). We report the results separately for the memory task and concurrent RT task.

#### Primary memory task

**Fig. 1:**
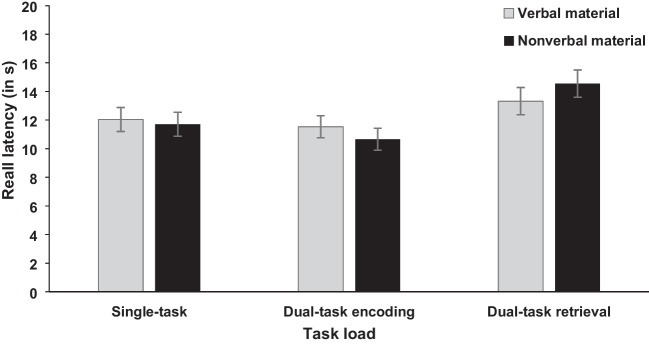
Mean recall latency (in s) of the memory task in Experiment 1. Error bars show standard errors

**Fig. 2:**
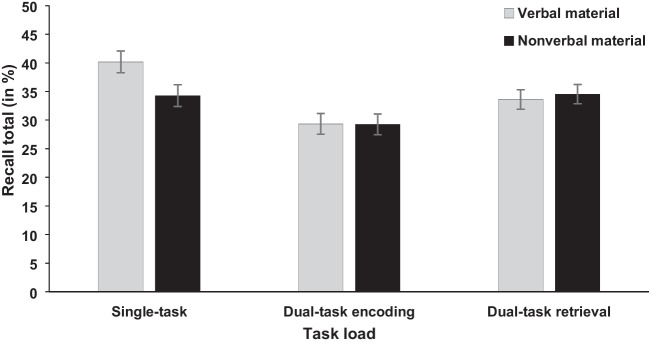
Mean recall total (in %) of the memory task in Experiment 1. Error bars show standard errors

The independent variables task load (single-task vs. dual-task encoding vs. dual-task retrieval) and stimulus material (arrow vs. word) were included in the analysis for recall latency and recall total (see Figs. 1 and 2). For task load, the ANOVA showed a significant main effect for recall latency, *F*(2, 92) = 8.91, *p* < .001, η_p_^2^ = .162, BF_10_ = 97.97, and recall total, *F*(2, 92) = 13.01, *p* < .001, η_p_^2^ = .220, BF_10_ = 3.32×10^3^. To further investigate the significant effect of task load on recall latency and recall total, we conducted one-tailed post hoc *t* tests (Bonferroni correction; also for the following post hoc *t* tests) and compared the single-task, dual-task at encoding, and dual-task at retrieval conditions.

Starting with the dual-task encoding and single-task condition, the *t* test yielded no significant effect for recall latency, *t*(47) = 1.13, *p* = .399, BF_10_ = 0.28; 11.09 sec (dual-task encoding) vs. 11.87 sec (single-task). However, for recall total, the test showed a significant difference, *t*(47) = 4.72, *p* < .001, *d*_z_ = 0.681, BF_10_ = 9.39×10^2^, indicating reduced recall total in the dual-task encoding compared with single-task condition (29.31% vs. 37.22%).

For the dual-task retrieval and single-task condition, the *t* test showed a significant difference for recall latency, *t*(47) = 2.97, *p* = .008, *d*_z_ = 0.429, BF_10_ = 7.36, with higher recall latency for the dual-task retrieval compared with single-task condition (13.94 sec vs. 11.87 sec). For recall total there was a non-significant trend shown by the frequentist analysis, but the Bayesian analysis indicated no favor for either the alternative or null hypothesis, *t*(47) = 2.11, *p* = .060, *d*_z_ = 0.305, BF_10_ = 1.19. The nonsignificant trend showed numerically reduced recall total for the dual-task retrieval compared with single-task condition (34.07% vs. 37.22%).

Lastly, for the dual-task retrieval and dual-task encoding conditions, the *t* test yielded a significant difference for recall latency, *t*(47) = 4.02, *p* < .001, *d*_z_ = 0.580, BF_10_ = 116.67, and recall total, *t*(47) = 2.98, *p* = .008, *d* = 0.430, BF_10_ = 7.50. Recall latency and recall total were higher in the dual-task retrieval compared with dual-task encoding condition (13.94 sec vs. 11.09 sec; 34.07% vs. 29.31%).

Further, the main effect of stimulus material was neither significant for recall latency, *F* < 1, BF_10_ = 0.27; 12.31 sec (arrow) vs. 12.30 sec (word), nor recall total, *F* < 1, BF_10_ = 0.36; 32.70% (arrow) vs. 34.40% (word). Also, the interaction between stimulus material and task load was not significant for recall latency, *F*(2, 92) = 1.23, *p* = .297, BF_10_ = 0.30 (see Fig. 1 for descriptive statistics) and only showed a nonsignificant trend for recall total by the frequentist analysis, which was not supported by the Bayesian analysis that indicated neither a favor for the alternative or null hypothesis, *F*(2, 92) = 2.80, *p* = .066, BF_10_ = 1.06, η_p_^2^ = .057 (see Fig. 2 for descriptive statistics).

#### Concurrent RT task

**Fig. 3:**
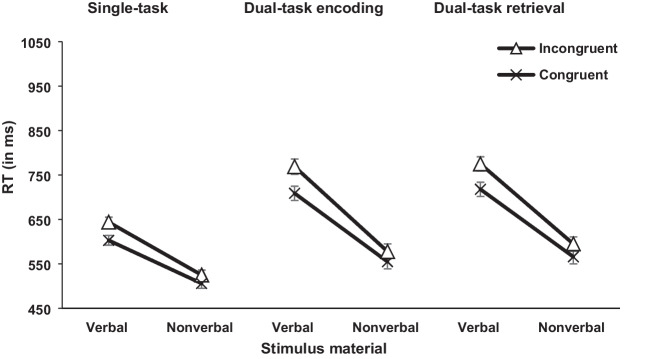
Mean RTs (in ms) of the concurrent RT task in Experiment 1. Error bars show standard errors

**Fig. 4:**
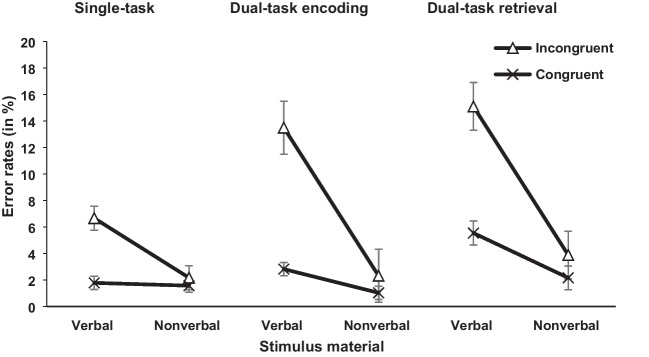
Mean error rates (in %) of the concurrent RT task in Experiment 1. Error bars show standard errors

The independent variables task load (single-task vs. dual-task encoding vs. dual-task retrieval), congruency (congruent vs. incongruent), and stimulus material (arrow vs. word) were included in the analysis for RT and error rates (see Figs. 3 and 4). For task load, there was a significant difference for RT, *F*(2, 92) = 55.59, *p* < .001, η_p_^2^ = .547, BF_10_ = 1.74×10^33^, and error rates, *F*(2, 92) = 11.09, *p* < .001, η_p_^2^ = .194, BF_10_ = 5.56×10^2^. To analyze the task load effects further, one-tailed post hoc *t* tests were conducted for RT and error rates comparing the single-task, dual-task encoding, and dual-task retrieval conditions.

Starting with the dual-task encoding and single-task condition, the *t* test showed significant higher RT in the dual-task encoding compared with single-task condition, *t*(47) = 8.26, *p* < .001, *d*_z_ = 1.192, BF_10_ = 9.67×10^7^; 651 ms vs. 569 ms. For error rates, the same pattern of results was shown by the post hoc *t* test, but this was not confirmed by the Bayesian analysis that showed neither support for the alternative or null hypothesis, *t*(47) = 2.25, *p* = .044, *d* = 0.324, BF_10_ = 1.53; 4.67% vs. 3.04%.

For the dual-task retrieval and single-task condition, the *t* tests yielded significant higher RT and error rates for the dual-task retrieval compared with single-task condition, RT, *t*(47) = 9.84, *p* < .001, *d*_z_ = 1.421, BF_10_ = 1.53×10^10^; 663 ms vs. 569 ms; error rates *t*(47) = 3.95, *p* < .001, *d*_z_ = 0.570, BF_10_ = 97.52; 6.69% vs. 3.04%.

Lastly, for the dual-task retrieval and dual-task encoding condition, there was no significant difference for RT, *t*(47) = 1.09, *p* = .423, BF_10_ = 0.27; 663 ms (dual-task retrieval) vs. 651 ms (dual-task encoding). However, results showed a significant effect for error rates, *t*(47) = 3.05, *p* = .006, *d*_z_ = 0.440, BF_10_ = 8.96, reflecting higher error rates in the dual-task retrieval compared with dual-task encoding condition (6.69% vs. 4.67%).

Further, the main effect of congruency was significant for RT, *F*(1, 46) = 153.38, *p* < .001, η_p_^2^ = .769, BF_10_ = 2.40×10^8^, and error rates, *F*(1, 46) = 34.46, *p* < .001, η_p_^2^ = .428, BF_10_ = 7.92×10^9^, with increased RT and error rates in incongruent compared with congruent trials (648 ms vs. 610 ms; 7.30% vs. 2.50%). Moreover, the interaction between task load and congruency showed a nonsignificant trend for RT by the frequentist analysis, but this was not supported by the Bayesian analysis which showed neither a favor for the alternative or null hypothesis, *F*(2, 92) = 2.99, *p* = .055, η_p_^2^ = .061, BF_10_ = 0.11; 43 ms (dual-task retrieval) vs. 41 ms (dual-task encoding) vs. 29 ms (single-task). For error rates, this interaction was significant in the frequentist analysis, *F*(2, 92) = 3.79, *p* = .026, η_p_^2^ = .076, BF_10_ = 0.65, but again not supported by the Bayesian analysis that showed no favor for either the alternative or null hypothesis. To analyze this interaction for error rates further, the congruency effects for the single-task, dual-task encoding and dual-task retrieval conditions were compared using post hoc *t* tests.

For the dual-task encoding and single-task condition, the *t* test showed a significant difference, *t*(47) = 2.56, *p* = .021, *d*_z_ = 0.370, BF_10_ = 2.91; 5.98% vs. 2.74%, reflecting a larger congruency effect in the dual-task encoding compared with the single-task condition. As previously, this was not supported by the Bayesian analysis which showed neither a favor for the alternative or null hypothesis.

For the dual-task retrieval and single-task condition the same pattern of result was shown by the frequentist analysis and Bayesian analysis, *t*(47) = 2.32, *p* = .038, *d*_z_ = 0.334, BF_10_ = 1.76; 5.64% vs. 2.74%, indicating a larger congruency effect for the dual-task retrieval compared with single-task condition.

Lastly, for the dual-task encoding and dual-task retrieval condition the *t* test showed no significant difference (*t* < 1, BF_10_ = 0.16; 5.98% [dual-task encoding] vs. 5.64% [dual-task retrieval]).

Moreover, for stimulus material there was a significant difference for RT, *F*(1, 46) = 82.90, *p* < .001, η_p_^2^ = .643, BF_10_ = 8.92×10^8^, and error rates, *F*(1, 46) = 28.20, *p* < .001, η_p_^2^ = .380, BF_10_ = 3.67×10^3^, indicating increased RT and error rates in the word group compared with the arrow group (703 ms vs. 554 ms; 7.60% vs. 2.20%). The interaction between stimulus material and congruency was significant for RT, *F*(1, 46) = 22.52, *p* < .001, η_p_^2^ = .329, BF_10_ = 4.62, and error rates, *F*(1, 46) = 19.35, *p* < .001, η_p_^2^ = .296, BF_10_ = 2.51 × 10^5^. The congruency effect was larger in the word group compared with the arrow group (52 ms vs. 23 ms; 8.40% vs. 1.60%). Also, the interaction between task load and stimulus material was significant for RT, *F*(2, 92) = 6.50, *p* = .002, η_p_^2^ = .124, BF_10_ = 7.93×10^3^, and error rates, *F*(2, 92) = 5.93, *p* = .004, η_p_^2^ = .114, BF_10_ = 8.96 (post hoc *t* tests for task load effects in both groups separately were conducted and can be found in the Appendix).

Lastly, the three-way interaction was not significant for RT, *F* < 1, BF_10_ = 0.14, or error rates, *F*(2, 92) = 2.05, *p* = .135, BF_10_ = 0.35.

### Discussion

Experiment 1 replicated the typical decrease in memory accuracy (with both arrows and words as stimulus material in the RT task) in the dual-task at encoding compared with single-task condition (e.g., Craik et al., 1996; Naveh-Benjamin et al., 2000a). For the dual-task at retrieval condition, we did not observe a significant decrease in memory accuracy compared with the single-task condition, also not with words as stimulus material in the RT task (contrary to the findings of Fernandes & Moscovitch, 2000), but recall latency was significantly increased.

Concurrent RT task performance was decreased for the dual-task at encoding and retrieval compared with single-task condition. Overall, performance of the word group was worse than for the arrow group. Additionally, the Stroop congruency effect in the word group was significantly larger compared with the arrow group. The Stroop congruency effect was also significantly larger for both dual-task conditions compared with the single-task condition, but please note that this was only significant for error rates (for RT there was only a nonsignificant trend shown by the frequentist analysis) and the Bayesian analysis was in the uninformative range and did not support these findings.

The decreased memory and concurrent RT task performance in the dual-task at encoding condition suggest that memory encoding processes require the same limited capacity as response selection in the concurrent RT task (e.g., Jolicoeur & Dell’Acqua, 1998). Thus, if both tasks require simultaneous access to a shared limited capacity, memory processes are slowed down due to parallel processing, resulting in a memory impairment in the primary memory task and a slower or more error prone response in the concurrent RT task. This could also explain the increased Stroop congruency effect in the dual-task at encoding compared with single-task condition shown by the frequentist analysis. The processing conflict (i.e., incongruent trials) in the concurrent RT task possibly placed larger demands on the shared limited capacity, resulting in even worse performance in incongruent trials in the dual-task compared with single-task condition. Thus, a possible relationship between processing conflicts in the concurrent RT task and success of memory encoding in the primary memory task was further investigated in Experiment 2.

For the dual-task at retrieval condition, the significantly increased recall latency compared with the single-task condition indicates that even if memory accuracy is not significantly decreased, dual-task costs are still present. We will further elaborate the effect on recall latency, as well as the contrary findings to Fernandes and Moscovitch (2000) on recall total in the general discussion.

## Experiment 2

The larger Stroop congruency effect in the concurrent RT task (shown by the frequentist analysis, but not supported by the Bayesian analysis) in the two dual-task conditions compared with the single-task condition indicates a possible relationship between memory processes and processing conflicts in the RT task. Experiment 2 investigated whether the processing conflict in the concurrent RT task modulates the success of memory encoding. Thus, the next step was to time-lock stimulus presentation of both tasks to analyze memory accuracy temporally linked to the occurrence of the processing conflict (i.e., incongruent trials) on a trial level. In this experiment, we focused on the memory encoding stage, not memory retrieval, because memory accuracy was not significantly affected in the latter condition. With this experimental design, we were able to examine if dual-task costs are influenced by general load or task-specific processing conflicts.

For Experiment 2, we hypothesized decreased memory accuracy and an impaired RT task performance in the dual-task condition at the memory encoding stage compared with the single-task settings like in Experiment 1. Additionally, the Stroop congruency effect in the RT task should be larger in the dual-task compared with single-task setting. Most importantly, we assumed decreased memory accuracy of items that were encoded when there is a high processing conflict (incongruent trials) in the RT task compared with items that were encoded during trials with a low processing conflict (congruent).

### Method

#### Stimuli, tasks, responses, and procedure

The stimuli, tasks, and responses were very similar to those in Experiment 1. A difference was that the arrows (symbolic stimulus material) were no longer used as visual stimuli for the RT task (i.e., spatial Stroop task), because we expected larger dual-task interference with the words (verbal stimulus material). Further, new auditory stimuli were created due to increased length of the word lists. The auditory stimuli consisted of 154 German three-syllable words, again mainly selected from the *Diagnostic and Statistical Manual of Mental Disorders* (American Psychiatric Association, 2013; e.g., “Amnesia,” “Dopamine,” “Empathy”). The same text-to-speech converter and voice as in Experiment 1 was used and the duration of each word was again adjusted to one second. Nine new lists with 16 words and two lists with five words were generated by randomly assigning the words to the lists with no word appearing in more than one list (word order per list randomized for each participant).

The procedure was slightly different from Experiment 1. There was only a dual-task condition at the encoding phase of the memory task, not the retrieval phase. Changes in the memory task were that the serial presentation of 16 words via headphones was now at a controlled rate of once per 3 sec, hence, the duration of the encoding phase was 48 seconds. For the RT task, the fixation cross and location stimulus were presented for up to 2,000 ms. The location stimulus disappeared after response execution or time-out (no response within 2,000 ms) and the fixation cross remained visible for a variable RSI of 3,000 ms minus RT to keep the time of one trial constant (3,000 ms). The error message was displayed for 300 ms, followed by the fixation cross for 2,700 ms minus RT (or minus 2,000 ms in case of a time-out). Participants performed 16 trials in each block (eight congruent and eight incongruent trials). These changes ensured that in the dual-task condition only one stimulus of the RT task was presented during the encoding of one word in the memory task (16 words in the memory task and 16 Stroop trials in total per block), so that stimulus presentation was time-locked across the tasks. In this way, we were able to analyze the effect of congruency in the RT task on encoding in the memory task on a trial level. The distractor activity and recall period of the memory task did not change.

Participants again performed a practice block of the RT task first (eight trials), followed by a practice block of the memory task (five-element word list, 20-sec encoding phase; 15-sec distractor activity; 20-sec recall period) and one dual-task practice block (five-element word list; five Stroop trials). In total, the experiment consisted of 12 experimental blocks (three single-task blocks of each the RT task and memory task; six dual-task blocks at the encoding stage of the memory task; counterbalanced with Latin square design across participants).

#### Design

For the primary memory task, we specified two different analyses. First, we tested the effects of task load (single-task vs. dual-task at encoding) on recall total and recall latency. Second, we examined the influence of congruency (congruent vs. incongruent) in the RT task on recall performance.

For the RT task, we analyzed performance based on a 2 × 2 repeated-measures design with the independent within-subject variables task load (single-task vs. dual-task at encoding) and congruency (congruent vs. incongruent). The dependent variables were RT and error rates.

#### Participants

Forty-five new recruited psychology students (35 female; mean age = 21.69 years, *SD* = 3.11) took part in the experiment. Again, they were pre-screened according to their age (between 18 and 35 years) and native language (German). All gave their informed consent for participation, reported normal or corrected-to-normal vision and hearing acuity, and received partial course credit or monetary compensation (10€ per hour) in exchange. Two additional participants were tested, but their data could not be analyzed due to exclusion criteria (see Results).

To determine the sample size, a power analysis was conducted using G*Power (Faul et al., 2007). For calculation, we focused on our most important hypothesis, which suggest that memory encoding is influenced by processing conflicts in the concurrent RT task. To this end, we calculated a simplified design comparing memory accuracy in the dual-task condition of words that were encoded during congruent trials vs. incongruent trials in the RT task, corresponding to a within-subjects *t* test. With a medium to small effect size (*d* = 0.4) and an alpha of 0.05, the power analysis showed that at least 41 participants were required to achieve a power of 0.8 (due to counterbalancing, 45 participants were recruited).

### Results

Data analysis proceeded like in Experiment 1,^2^ with the same criteria for exclusion (*n* = 2) and outliers (5.00% errors and 0.40% outliers excluded). We report the results separately for the memory task and concurrent RT task.

#### Primary memory task

**Fig. 5:**
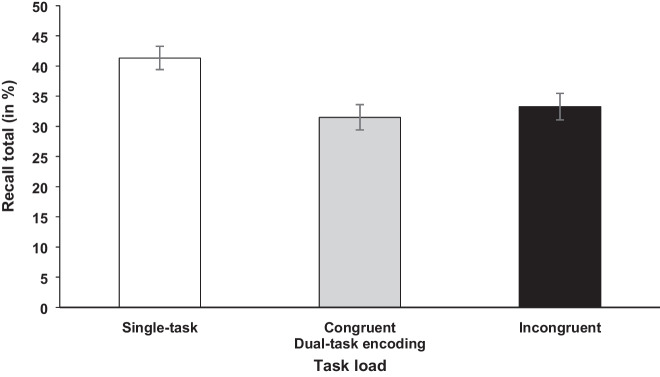
Mean recall total (in %) of the memory task in Experiment 2. Error bars show standard errors

The data were submitted to *t* tests with the independent variable task load (single-task vs. dual-task encoding) for recall latency and recall total. For recall latency, there was no significant difference (*t* < 1, BF_10_ = 0.25; 12.08 sec [dual-task encoding] vs. 12.65 sec [single-task]). However, for recall total, the test showed a significant difference, *t*(44) = 7.43, *p* < .001, *d* = 1.110, BF_10_ = 4.38×10^6^, indicating reduced recall total for the dual-task encoding compared with single-task condition (32.39% vs. 41.34%; see Fig. 5).

Further, only for the dual-task encoding blocks, data was submitted to a *t* test with the independent variable congruency (congruent vs. incongruent) for recall total. The results showed no significant difference in recall performance between words that were encoded in congruent (31.50%) compared with incongruent (33.29%) trials of the concurrent RT task, *t*(44) = 1.20, *p* = .118, BF_10_ = 0.29, BF_01_ = 3.43 (see Fig. 5).^3^

#### Concurrent RT task

**Fig. 6:**
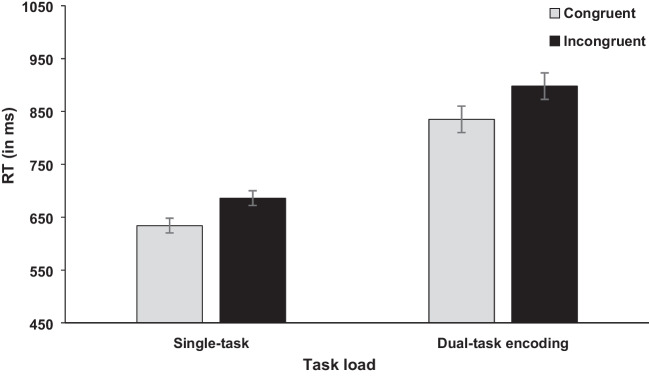
Mean RTs (in ms) of the concurrent RT task in Experiment 2. Error bars show standard errors

**Fig. 7:**
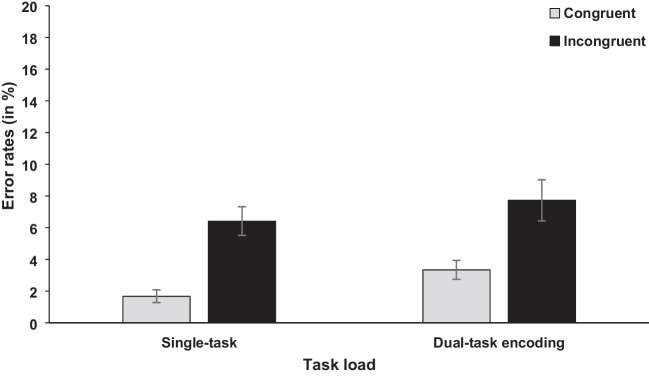
Mean error rates (in %) of the concurrent RT task in Experiment 2. Error bars show standard errors

The independent variables task load (single-task vs. dual-task encoding) and congruency (congruent vs. incongruent) were included in the analysis for RT and error rates (see Figs. 6 and 7). For task load, the analysis showed a significant difference for RT, *F*(1, 44) = 103.64, *p* < .001, η_p_^2^ = .702, BF_10_ = 1.28×10^68^, reflecting higher RTs for the dual-task encoding compared with single-task condition (866 ms vs. 660 ms). For error rates, the main effect was not significant, *F*(1, 44) = 2.79, *p* = .102, BF_10_ = 1.89; 5.50% (dual-task encoding) vs. 4.00% (single-task), but the data pattern was numerically in the same direction.

Further, the main effect of congruency was significant for RT, *F*(1, 44) = 125.28, *p* < .001, η_p_^2^ = .740, BF_10_ = 4.74×10^7^, and error rates, *F*(1, 44) = 33.59, *p* < .001, η_p_^2^ = .433, BF_10_ = 5.47×10^9^, with higher RTs and error rates in incongruent compared with congruent trials (792 ms vs. 734 ms; 7.00% vs. 2.50%). The interaction between task load and congruency was not significant, RT,* F*(1, 44) = 2.34, *p* = .133, BF_10_ = 0.20; error rates,* F* < 1, BF_10_ = 0.16.

### Discussion

In Experiment 2, we again showed the typical decrease in memory accuracy of the primary memory task and higher RTs in the concurrent RT task due to dual-task interference (e.g., Craik et al., 1996). Further, we found a Stroop congruency effect in the concurrent RT task. Yet, in the RT task, we did not observe an increased Stroop congruency effect in the dual-task at encoding condition compared with the single-task condition (like in Experiment 1). In addition, we did not find a significant impact of the Stroop congruency effect on the primary memory task.

The findings again implicate that memory encoding processes interfere with response selection in the RT task (e.g., Jolicoeur, 1999a, 1999b). We could not find evidence for a direct relationship between the success of memory encoding in the primary memory task and the occurrence of processing conflicts in the concurrent RT task, which was also supported by the Bayesian analysis that favors the null hypothesis over the alternative hypothesis. Based on the results we cautiously assume that memory encoding is primarily influenced by general load (due to parallel processing with the concurrent RT task and a shared limited capacity) instead of task-specific processing conflicts.

## General discussion

In the present study, we aimed to investigate the detrimental effects of dual-task demands on memory encoding and retrieval of auditory information. To this end, we conducted an experiment with a dual-task condition at either the encoding or the retrieval stage of a free-recall memory task and different stimulus material (arrows vs. words) in a concurrent RT task (i.e., a spatial Stroop task; Experiment 1). Further, we were interested in the relationship of successful memory encoding in the primary memory task and the occurrence of processing conflicts (i.e., Stroop congruency effect) in the concurrent RT task. To investigate this, in Experiment 2 we time-locked stimulus presentation of the memory and RT task to analyze the impact of processing conflicts in the RT task on memory encoding in the primary memory task on a trial level. Note that to our knowledge, this is the first time that in a free recall memory task the success of memory encoding is temporally linked to processing conflicts in a RT task.

## Differences between memory encoding and retrieval processes

In both experiments in the primary memory task, we found clear dual-task interference at memory encoding on recall performance (see, e.g., Anderson & Craik, 1974; Baddeley et al., 1984; Craik et al., 1996; Fernandes & Moscovitch, 2000; Naveh-Benjamin et al., 2000a). In addition, performance in the concurrent RT task was impaired in the dual-task condition at memory encoding. This fits to the idea that encoding processes in the primary memory task interferes with response selection in the RT task, because both processes compete for a shared limited capacity. We assume that the parallel processing results in slower processing speed in both tasks and thus, impairments of the memory trace and slower or more error prone responses in the RT task (Jolicoeur, 1999a, 1999b; Jolicoeur & Dell’Acqua, 1998; Koch & Jolicoeur, 2007; Koch et al., 2003; Koch & Prinz, 2002; Koch & Prinz, 2005).

For the dual-task condition at memory retrieval, we did not observe a significant decline in recall performance, but participants recalled the words more slowly and concurrent RT task performance was impaired. These results are consistent with findings reported by Craik et al. (2018). In their second experiment, they manipulated the relative emphasis between a recognition memory task and a RT task at memory retrieval. In the memory encoding phase, 12 word pairs were presented and half of them re-paired for the recognition phase (i.e., six word pairs were still intact, six word pairs were randomly re-paired). Participants were then asked to judge whether the word pairs were intact or re-paired, in a single- or dual-task condition with different emphasis conditions (memory or RT task more important, or 50:50). Results showed that response rates of both tasks trade off against each other in the different emphasis conditions, while recognition accuracy remained constant. They concluded that retrieval is attention demanding, but accuracy is maintained by a compensatory increase in decision latency, thus, there is a trade-off within the recognition task itself. The authors assumed that participants prioritized accuracy over time, by accrue evidence for their decision until some criterion of confidence is satisfied, but this response decision process is slowed down due to parallel processing with the response selection in the concurrent RT task.

We did not find results similar to those reported by Fernandes and Moscovitch (2000). They observed that recall performance in the dual-task at memory retrieval condition was modulated by the degree to which processing resources overlap (i.e., both tasks include verbal processing). Notably, in the present Experiment 1 performance of the concurrent RT task was worse in the word group compared with arrow group in general, possibly due to the overlap of verbal resources also needed for the primary memory task. In addition, the Stroop congruency effect was larger in the word group compared with the arrow group. Thus, one possible explanation could be that the effect of overlapping processing resources did not affect memory performance in the primary memory task but concurrent RT task performance, because participants allocated their attentional resources more to the memory task. Note that it could also be that words as stimuli for the RT task are simply more difficult to process than arrows and thus, concurrent RT task performance was worse in the word group compared with arrow group.

## Success of memory encoding and processing conflicts in the concurrent RT task

In Experiment 1, in the RT task we found a significantly increased Stroop congruency effect in the dual-task encoding condition compared with the single task condition, indicating an increased processing conflict. Note that this effect was only present in the frequentist analysis for error rates (for RTs there was a nonsignificant trend) and not supported by the Bayesian analysis that showed neither support for the alternative nor the null hypothesis. In Experiment 2, we found a Stroop congruency effect in the RT task, but there was no significant difference between the dual-task encoding and single-task condition. Additionally and most importantly, we did not find a significant impact of processing conflicts in incongruent Stroop trials on success of memory encoding in the primary memory task, which was further supported by the Bayesian analysis.

Since incongruent trials are associated with longer RTs and higher error rates due to the processing conflict, we predicted worse memory accuracy in those trials due to possibly larger demands on the shared limited capacity. As mentioned earlier, we assume that encoding processes in the primary memory task are slowed down and impaired due to parallel processing with the concurrent RT task. However, it seems that this interference is primarily influenced by general load and not task-specific processing conflicts in the concurrent RT task.

Another possible explanation for our result that we did not find a significant difference in successful memory encoding in congruent and incongruent Stroop trials could be that participants used different encoding strategies across trials, meaning that they allocated their attentional resources differently in trials with low and high processing conflicts. A line of research investigating selective memory could support this idea. In a study, Middlebrooks et al. (2017) examined the impact of dual-tasks on the ability to selectively remember valuable information. In Experiment 1, participants either studied visually presented words with a value from 1 to 10 points in a single-task condition, while listening to music or completing a digit-detection task. Results showed that while performing a concurrent digit-detection task at the memory encoding stage, recall performance was impaired in general, but there was no significant difference in the number of recalled high-value words in contrast to the other conditions. Similar results were found in Experiment 2 with different concurrent tasks, with varying difficulty and demands on working memory (three different tone-detection tasks; tone-monitoring, paired-tones, and 1-back). The authors concluded that concurrent tasks, which compete for the shared limited capacity and impair memory, do not necessarily impair encoding strategies. However, recent work by Murphy et al. (2024) did not confirm these results (see also Elliott & Brewer, 2019; Murphy & Castel, 2023). In their study, the authors explored the impact of dual-tasks at memory encoding and retrieval on memory selectivity and found that reducing attentional resources at the encoding stage impairs memory for valuable information (in contrast, dual-tasks at the retrieval stage did not impair selective memory). Important to mention is that in Experiment 1a, they used the same concurrent task as in the study of Middlebrooks et al. (2017). They suggested that participants may allocate their attentional resources in a different manner across trials and further research is needed to detect possible moderating factors.

### Insights for applied scenarios

For applied contexts, like the medical scenarios described earlier in the Introduction, our results suggest that when performing a memory encoding and concurrent task in parallel, performance in both tasks will suffer. That means that not only the information loss could lead to negative consequences in the context of handover patient-related information in health care, but also the probability for errors is increasing. This is especially the case if the simultaneous task includes processing of similar material or information compared with auditory information that needs to be encoded. Second, it seems that regardless of whether the tasks include some kind of decision conflicts or not, in both cases memory encoding processes will be disturbed. Thus, to reduce information loss and to facilitate task performance, support systems (e.g., automatic speech recognition systems) are desirable that reduce the probability that medical staff needs to encode auditory information and perform other tasks in parallel.

### Limitations and future research

One possible limitation of this study could be that the temporal density of the concurrent RT task we used in Experiment 2 was too low to find an effect of processing conflicts in the RT on memory encoding in the primary memory task. In order to time-lock stimulus presentation of both tasks, we increased the time between stimulus presentations in Experiment 2 from a 2,000 ms interval to a 3,000 ms interval. Rohrer and Pashler (2003) argued that the more time participants spend on response selection processes the greater the demands on “central” capacity (i.e., capacity for memory processes). Possibly, because participants had more time for processing in the concurrent RT task, they carried out processing operations for the primary memory task first, especially in incongruent trials. However, we still observed a decreased memory accuracy in general, indicating that both tasks competed for a shared limited capacity.

Another limitation of this study could be a lack of power. With the calculated power, we were only able to detect medium effects in Experiment 1. In addition, Brysbaert and Stevens (2018) recently suggested that a properly powered experiment with repeated measures should have at least 1,600 observations per condition (e.g., 40 participants and 40 trials per condition). In Experiment 1, 24 (48/2 due to between-subjects factor) participants performed 45 trials per condition (three blocks with 15 stimuli per condition), resulting in 1,080 observations. Thus, based on Brysbaert and Stevens’s (2018) calculation, Experiment 1 is slightly underpowered.

However, in Experiment 2, we were able to detect medium to small effects with the calculated power. Moreover, according to the calculation of Brysbaert and Stevens (2018), 45 participants completed 48 trials per condition in the dual-task blocks (six blocks with eight stimuli per condition), resulting in 2,160 observations. With this power, we are confident that the null effect of successful memory encoding and the occurrence of processing conflicts in the RT task is not caused due to a lack of power.

For future research it would be interesting to manipulate the SOA and sequence of the stimuli in both tasks (i.e., memory stimulus first or RT stimulus first, with different time between stimulus presentation), to investigate the source of interference between memory encoding processes in a free recall memory task and processing conflicts in a RT task more deeply.

## Conclusion

In sum**,** in two dual-task experiments with a free recall memory task and a concurrent RT task (i.e., a spatial Stroop task), we found clear evidence for strong dual-task interference at the memory encoding as well as retrieval stage. We assume that this interference occurred because memory processes and response selection in the RT task share the same limited capacity. Capacity sharing results in slower processing speed in both tasks, in turn resulting in a decreased memory and concurrent RT task performance. Further, in the dual-task conditions we observed increased processing conflicts in the concurrent RT task. However, we could not find significant evidence for a relationship between processing conflicts in the concurrent RT task or success of memory encoding in the primary memory task. Thus, we assume that the dual-task interference primarily occurs due to general load instead of task-specific processing conflicts.

## Data Availability

The datasets generated and analyzed during the current study are available online (https://osf.io/s3eby/?view_only=4a4d7d7050144eb6acc682260bed14d0). Preregistration protocol of Experiment 2 is available (https://osf.io/jvzxn). There was no preregistration for Experiment 1.

## Appendix

### Post hoc *t* tests for interaction between task load and stimulus material in the RT task

In Experiment 1, we found a significant interaction between task load and stimulus material for RT and error rates in the concurrent RT task. Results are described here, in the appendix, since our hypothesis focused on the material specific effects (arrow vs. word) on recall performance in the primary memory task and not concurrent RT task performance. To analyze the task load effects in both groups, post hoc *t* tests were conducted comparing the single-task, dual-task encoding, and dual-task retrieval conditions for RT and error rates separately for the arrow and word group.

Starting with the dual-task encoding and single-task condition, the *t* test showed significant increased RTs in the dual-task encoding compared with the single-task condition in both the arrow and word groups. The same pattern of results was shown for the dual-task retrieval compared with single-task condition in both groups (all *t* > 4.32, *p* < .001, *d*_z_ ≥ 0.884 and BF_10_ ≥ 121.36; for descriptive statistics, see Fig. 3). In addition, there was no significant difference for RT between the dual-task encoding and dual-task retrieval condition in both groups (*t* < 1 and BF_10_ ≤ 0.27).

For error rates in the arrow group, neither the difference between the dual-task encoding and single-task condition (*t* < 1; 1.65% vs. 1.88%, BF_10_ = 0.26) nor the differences between the dual-task retrieval and single-task condition, *t*(23) = 1.81, *p* = .126; 3.04% vs. 1.88%, BF_10_ = 0.25, was significant. However, the *t* test showed a significant difference between the dual-task encoding and dual-task retrieval conditions, *t*(23) = 2.63, *p* = .023, *d*_z_ = 0.537, BF_10_ = 3.47, indicating higher error rates for the dual-task retrieval compared with the dual-task encoding condition (3.04% vs. 1.65%).

For error rates in the word group, results showed significant differences between the dual-task encoding and single-task condition, *t*(23) = 2.65, *p* = .021, *d*_z_ = 0.540, BF_10_ = 3.55; 7.69% vs. 4.20%, and dual-task retrieval and single-task condition, *t*(23) = 3.85, *p* = .002, *d*_z_ = 0.786, BF_10_ = 3.56; 10.33% vs. 4.20%, reflecting higher error rates in both dual-task compared with single-task conditions. Additionally, there was a nonsignificant trend for the dual-task retrieval and dual-task encoding conditions shown by the frequentist analysis, but the Bayesian analysis indicated favor for neither the alternative nor the null hypothesis, *t*(23) = 2.18, *p* = .060, *d*_z_ = 0.444, BF_10_ = 1.56. The nonsignificant trend indicated numerically higher error rates for the dual-task retrieval compared with the dual-task encoding condition (10.33% vs. 7.69%).

Varying performance in the concurrent RT task due to the different stimulus material (arrow vs. word) is further discussed in the General Discussion.

## Footnotes

1 Originally, we wanted to control for the working memory capacity between both groups because it has been identified as a crucial construct for variance in dual-task performance (Redick et al., 2016). However, studies have shown that the digit span task is not suitable for assessing working memory capacity, as it measures a short-term memory capacity that only involves a storage component. Instead, a complex span task should have been used as it measures both the storage and processing of information (see, e.g., Redick et al., 2012).

2 Note that the Bayesian analysis was not preregistered.

3 As exploratory analysis, we aimed to control for the congruency sequence effect (CSE; Gratton et al., 1992). The CSE denotes the finding that the congruency effect is smaller when the previous trial was incongruent compared with congruent due to conflict-driven adjustments in cognitive control (Botvinick et al., 2001). If the occurrence of processing conflicts in the RT task has an influence on the success of memory encoding, memory accuracy of items that are encoded in congruent following congruent trials (low processing conflict) should be higher than for items encoded in incongruent following congruent trials (high processing conflict without increased cognitive control). However, results showed no significant differences in recall performance.

## Code availability

The experimental codes and stimuli from the current study are available from the corresponding author on reasonable request.

## Declarations

### Open practice statement

The datasets generated and analyzed during the current study are available online (https://osf.io/s3eby/?view_only=4a4d7d7050144eb6acc682260bed14d0). Preregistration protocol of Experiment 2 is available (https://osf.io/jvzxn). There was no preregistration for Experiment 1.

## References

[CR1] American Psychiatric Association. (2013). *Diagnostic and statistical manual of mental disorders—5th edition revised (DSM-5)*. Washington DC: APA.

[CR2] Anderson, C. M. B., & Craik, F. I. M. (1974). The effect of a concurrent task on recall from primary memory. *Journal of Verbal Learning and Verbal Behavior,**13*, 107–113. 10.1016/S0022-5371(74)80035-6

[CR3] Baddeley, A. (2000). The episodic buffer: A new component of working memory? *Trends in Cognitive Sciences,**4*, 417–423. 11058819 10.1016/S1364-6613(00)01538-2

[CR4] Baddeley, A., & Hitch, G. (1974). Working memory. In G.A. Bower (Ed.), *Recent advances in learning and motivation* (Vol. 8, pp. 47–90). Academic Press.

[CR5] Baddeley, A., Lewis, V., Eldridge, M., & Thomson, N. (1984). Attention and retrieval from long-term memory. *Journal of Experimental Psychology: General,**113*, 518–540. 10.1037/0096-3445.113.4.518

[CR6] Baddeley, A., Thomson, N., & Buchanan, M. (1975). Word length and the structure of short-term memory. *Journal of Verbal Learning and Verbal Behavior,**14*, 575–589. 10.1016/S0022-5371(75)80045-4

[CR7] Botvinick, M. M., Braver, T. S., Barch, D. M., Carter, C. S., & Cohen, J. D. (2001). Conflict monitoring and cognitive control. *Psychological Review,**108*, 624–652. 11488380 10.1037/0033-295X.108.3.624

[CR8] Brysbaert, M., & Stevens, M. (2018). Power analysis and effect size in mixed effects models: A tutorial. *Journal of Cognition, 1*, Article 9. 10.5334/joc.10 PMC6646942 31517183

[CR9] Campbell, J. I. D., & Thompson, V. A. (2012). MorePower 6.0 for ANOVA with relational confidence intervals and Bayesian analysis. *Behavior Research Methods,**44*, 1255–1265. 22437511 10.3758/s13428-012-0186-0

[CR10] Craik, F. I. M., Eftekhari, E., & Binns, M. A. (2018). Effects of divided attention at encoding and retrieval: Further data. *Memory & Cognition,**46*, 1263–1277. 29934748 10.3758/s13421-018-0835-3

[CR11] Craik, F. I. M., Govoni, R., Naveh-Benjamin, M., & Anderson, N. D. (1996). The effects of divided attention on encoding and retrieval processes in human memory. *Journal of Experimental Psychology: General,**125*, 159–180. 8683192 10.1037/0096-3445.125.2.159

[CR12] Draheim, C., Pak, R., Draheim, A. A., & Engle, R. W. (2022). The role of attention control in complex real-world tasks. *Psychonomic Bulletin & Review,**29*, 1143–1197. 35167106 10.3758/s13423-021-02052-2 PMC8853083

[CR13] Elliott, B. L., & Brewer, G. A. (2019). Divided attention selectively impairs value-directed encoding. *Collabra: Psychology, 5*, Article 4.

[CR14] Evans, S. M., Murray, A., Patrick, I., Fitzgerald, M., Smith, S., Andrianopoulos, N., & Cameron, P. (2010). Assessing clinical handover between paramedics and the trauma team. *Injury,**41*, 460–464. 19735917 10.1016/j.injury.2009.07.065

[CR15] Fagot, C., & Pashler, H. (1992). Making two responses to a single object: Implications for the central attentional bottleneck. *Journal of Experimental Psychology: Human Perception and Performance,**18*, 1058–1079. 1431744 10.1037//0096-1523.18.4.1058

[CR16] Faul, F., Erdfelder, E., Lang, A.-G., & Buchner, A. (2007). G*Power 3: A flexible statistical power analysis program for the social, behavioral, and biomedical sciences. *Behavior Research Methods,**39*, 175–191. 17695343 10.3758/BF03193146

[CR17] Fernandes, M. A., & Moscovitch, M. (2000). Divided attention and memory: Evidence of substantial interference effects at retrieval and encoding. *Journal of Experimental Psychology: General,**129*, 155–176. 10868332 10.1037/0096-3445.129.2.155

[CR18] Fernandes, M. A., & Moscovitch, M. (2002). Factors modulating the effect of divided attention during retrieval of words. *Memory & Cognition,**30*, 731–744. 12219890 10.3758/BF03196429

[CR19] Fernandes, M. A., & Moscovitch, M. (2003). Interference effects from divided attention during retrieval in younger and older adults. *Psychology and Aging,**18*, 219–230. 12825772 10.1037/0882-7974.18.2.219

[CR20] Fischer, R., & Janczyk, M. (2022). Dual-task performance with simple tasks. In A. Kiesel, L. Johannsen, I. Koch, & H. Müller (Eds.), *Handbook of human multitasking* (pp. 3–36). Springer International Publishing.

[CR21] Gratton, G., Coles, M. G., & Donchin, E. (1992). Optimizing the use of information: Strategic control of activation of responses. *Journal of Experimental Psychology: General,**121*, 480–506. 1431740 10.1037/0096-3445.121.4.480

[CR22] Heng, K. W. (2014). Teaching and evaluating multitasking ability in emergency medicine residents—What is the best practice? *International Journal of Emergency Medicine,**7*, 1–5. 25635201 10.1186/s12245-014-0041-4 PMC4306081

[CR23] Jolicoeur, P. (1999). Concurrent response-selection demands modulate the attentional blink. *Journal of Experimental Psychology: Human Perception and Performance,**25*, 1097–1113. 10.1037//0096-1523.25.6.1483 10681214

[CR24] Jolicoeur, P. (1999). Dual-task interference and visual encoding. *Journal of Experimental Psychology: Human Perception and Performance,**25*, 596–616.

[CR25] Jolicoeur, P., & Dell’Acqua, R. (1998). The demonstration of short-term consolidation. *Cognitive Psychology,**36*, 138–202. 9721199 10.1006/cogp.1998.0684

[CR26] Kahnemann, D. (1973). *Attention and effort*. Englewood Cliffs: Prentice-Hall.

[CR27] Kass, R. E., & Raftery, A. E. (1995). Bayes factors. *Journal of the American Statistical Association,**90*, 773–795. 10.1080/01621459.1995.10476572

[CR28] Koch, I., & Jolicoeur, P. (2007). Orthogonal cross-task compatibility: Abstract spatial coding in dual tasks. *Psychonomic Bulletin & Review,**14*, 45–50. 17546730 10.3758/BF03194026

[CR29] Koch, I., Metin, B., & Schuch, S. (2003). The role of temporal unpredictability for process interference and code overlap in perception-action dual tasks. *Psychological Research,**67*, 244–252. 12684780 10.1007/s00426-002-0125-2

[CR30] Koch, I., Poljac, E., Müller, H., & Kiesel, A. (2018). Cognitive structure, flexibility, and plasticity in human multitasking - An integrative review of dual-task and task-switching research. *Psychological Bulletin,**144*, 557–583. 29517261 10.1037/bul0000144

[CR31] Koch, I., & Prinz, W. (2002). Process interference and code overlap in dual-task performance. *Journal of Experimental Psychology: Human Perception and Performance,**28*, 192–201.

[CR32] Koch, I., & Prinz, W. (2005). Response preparation and code overlap in dual tasks. *Memory & Cognition,**33*, 1085–1095. 16496728 10.3758/BF03193215

[CR33] Lozito, J. P., & Mulligan, N. W. (2006). Exploring the role of attention during memory retrieval: Effects of semantic encoding and divided attention. *Memory & Cognition,**34*, 986–998. 17128598 10.3758/BF03193246

[CR34] Lu, C. H., & Proctor, R. W. (1995). The influence of irrelevant location information on performance: A review of the Simon and spatial Stroop effects. *Psychonomic Bulletin & Review,**2*, 174–207. 24203654 10.3758/BF03210959

[CR35] McCann, R. S., & Johnston, J. C. (1992). Locus of the single-channel bottleneck in dual-task interference. *Journal of Experimental Psychology: Human Perception and Performance,**18*, 471–484.

[CR36] Middlebrooks, C. D., Kerr, T., & Castel, A. D. (2017). Selectively distracted: Divided attention and memory for important information. *Psychological Science,**28*, 1103–1115. 28604267 10.1177/0956797617702502 PMC5546942

[CR37] Murdock, B. B. (1965). Effects of a subsidiary task on short-term memory. *British Journal of Psychology,**56*, 413–419. 10.1111/j.2044-8295.1965.tb00983.x

[CR38] Murphy, D. H., & Castel, A. D. (2023). Responsible attention: The effect of divided attention on metacognition and responsible remembering. *Psychological Research,**87*, 1085–1100. 35838835 10.1007/s00426-022-01711-w PMC10191991

[CR39] Murphy, D. H., Schwartz, S. T., & Castel, A. D. (2024). Value-directed retrieval: The effects of divided attention at encoding and retrieval on memory selectivity and retrieval dynamics. *Journal of Experimental Psychology: Learning, Memory, and Cognition.,**50*(1), 17–38. 10.1037/xlm0001264 37326541 10.1037/xlm0001264

[CR40] Naveh-Benjamin, M., Craik, F. I., Gavrilescu, D., & Anderson, N. D. (2000a). Asymmetry between encoding and retrieval processes: Evidence from divided attention and a calibration analysis. *Memory & Cognition,**28*, 965–976. 11105522 10.3758/BF03209344

[CR41] Naveh-Benjamin, M., Craik, F. I., Perretta, J. G., & Tonev, S. T. (2000b). The effects of divided attention on encoding and retrieval processes: The resiliency of retrieval processes. *The Quarterly Journal of Experimental Psychology,**53*, 609–625. 10994220 10.1080/713755914

[CR42] Naveh-Benjamin, M., Guez, J., Hara, Y., Brubaker, M. S., & Lowenschuss-Erlich, I. (2014). The effects of divided attention on encoding processes under incidental and intentional learning instructions: Underlying mechanisms? *The Quarterly Journal of Experimental Psychology,**67*, 1682–1696. 24283628 10.1080/17470218.2013.867517

[CR43] Naveh-Benjamin, M., Kilb, A., & Fisher, T. (2006). Concurrent task effects on memory encoding and retrieval: Further support for an asymmetry. *Memory & Cognition,**34*, 90–101. 16686109 10.3758/BF03193389

[CR44] Pashler, H. (1994). Dual-task interference in simple tasks: Data and theory. *Psychological Bulletin,**116*, 220–244. 7972591 10.1037/0033-2909.116.2.220

[CR45] Peirce, J. W., Gray, J. R., Simpson, S., MacAskill, M. R., Höchenberger, R., Sogo, H., Kastman, E., & Lindeløv, J. (2019). PsychoPy2: Experiments in behavior made easy. *Behavior Research Methods,**51*, 195–203. 30734206 10.3758/s13428-018-01193-y PMC6420413

[CR46] Ramsay, M. C., & Reynolds, C. R. (1995). Separate digits tests: A brief history, a literature review, and a reexamination of the factor structure of the Test of Memory and Learning (TOMAL). *Neuropsychology Review,**5*, 151–171. 8653107 10.1007/BF02214760

[CR47] Rohrer, D., & Pashler, H. E. (2003). Concurrent task effects on memory retrieval. *Psychonomic Bulletin & Review,**10*, 96–103. 12747495 10.3758/BF03196472

[CR48] Redick, T. S., Broadway, J. M., Meier, M. E., Kuriakose, P. S., Unsworth, N., Kane, M. J., & Engle, R. W. (2012). Measuring working memory capacity with automated complex span tasks. *European Journal of Psychological Assessment,**28*, 164–171. 10.1027/1015-5759/a000123

[CR49] Redick, T. S., Shipstead, Z., Meier, M. E., Montroy, J. J., Hicks, K. L., Unsworth, N., Kane, M. J., Hambrick, D. Z., & Engle, R. W. (2016). Cognitive predictors of a common multitasking ability: Contributions from working memory, attention control, and fluid intelligence. *Journal of Experimental Psychology: General,**145*, 1473–1492. 27797557 10.1037/xge0000219

[CR50] Rouder, J. N., Morey, R. D., Verhagen, J., Swagman, A. R., & Wagenmakers, E.-J. (2017). Bayesian analysis of factorial designs. *Psychological Methods,**22*, 304–321. 27280448 10.1037/met0000057

[CR51] Skinner, E. I., & Fernandes, M. A. (2008). Interfering with remembering and knowing: Effects of divided attention at retrieval. *Acta Psychologica,**127*, 211–221. 17599796 10.1016/j.actpsy.2007.05.001

[CR52] Welford, A. T. (1952). The psychological refractory period and the timing of high-speed performance—A review and a theory. *British Journal of Psychology,**43*, 2–19.

